# Phylogenetic and transcriptional analysis of an expanded bZIP transcription factor family in *Phytophthora sojae*

**DOI:** 10.1186/1471-2164-14-839

**Published:** 2013-11-28

**Authors:** Wenwu Ye, Yang Wang, Suomeng Dong, Brett M Tyler, Yuanchao Wang

**Affiliations:** Department of Plant Pathology, Nanjing Agricultural University, Nanjing, 210095 China; Center for Genome Research and Biocomputing, and Department of Botany and Plant Pathology, Oregon State University, Corvallis, OR 97331 USA

**Keywords:** *Phytophthora*, bZIP transcription factor, DNA-binding domain, Duplication, Gene expression

## Abstract

**Background:**

Basic leucine zipper (bZIP) transcription factors are present exclusively in eukaryotes and constitute one of the largest and most diverse transcription factor families. The proteins are responsible for central developmental and physiological processes in plants, animals, and fungi, including the pathogenicity of fungal plant pathogens. However, there is limited understanding of bZIPs in oomycetes, which are fungus-like organisms in the kingdom Stramenopila. Oomycetes include many destructive plant pathogens, including the well-studied species *Phytophthora sojae*, which causes soybean stem and root rot.

**Results:**

Candidate bZIPs encoded in the genomes of *P. sojae* and four other oomycetes, two diatoms, and two fungal species were predicted using bioinformatic methods. Comparative analysis revealed expanded numbers of bZIP candidates in oomycetes, especially the *Phytophthora* species, due to the expansion of several novel bZIP classes whose highly conserved asparagines in basic DNA-binding regions were substituted by other residues such as cysteine. The majority of these novel bZIP classes were mostly restricted to oomycetes. The large number of novel bZIPs appears to be the result of widespread gene duplications during oomycete evolution. The majority of *P. sojae* bZIP candidates, including both conventional and novel bZIP classes, were predicted to contain canonical protein secondary structures. Detection of gene transcripts using digital gene expression profiling and qRT-PCR suggested that most of the candidates were not pseudogenes. The major transcriptional shifts of bZIPs occurred during the zoosporangia/zoospore/cyst and host infection stages. Several infection-associated bZIP genes were identified that were positively regulated by H_2_O_2_ exposure.

**Conclusions:**

The identification of large classes of bZIP proteins in oomycetes with novel bZIP motif variants, that are conserved and developmentally regulated and thus presumably functional, extends our knowledge of this important family of eukaryotic transcription factors. It also lays the foundation for detailed studies of the roles of these proteins in development and infection in *P. sojae* and other oomycetes.

**Electronic supplementary material:**

The online version of this article (doi:10.1186/1471-2164-14-839) contains supplementary material, which is available to authorized users.

## Background

Transcription factors (TFs) are key nodes of regulatory networks that bind to the promoter regions of target genes and regulate transcription. The basic leucine zipper (bZIP) family of TFs is one of the largest and most diverse TF families found exclusively in eukaryotes. bZIPs can be identified based on the bZIP domain, which is 60 to 80 amino acids in length and consists of a basic region and a leucine zipper [1]. The basic region is highly conserved and consists of approximately 16 amino acid residues with an invariant N-×7-R/K domain for nuclear localization and DNA binding. The leucine zipper mediates homo- and/or heterodimerization of bZIP proteins and is less conserved, consisting of a heptad repeat of leucine or other bulky hydrophobic amino acids, which is positioned exactly nine amino acids toward the C-terminus [1]. When bound to DNA, bZIP monomers are long α-helices; the N-terminal half of the basic region inserts into the major groove of double-stranded DNA where it binds to specific sequences, while the C-terminal half of the leucine zipper mediates dimerization to form a superimposed coiled-coil structure [2–4].

The bZIP family is multifunctional across species, and in fungal plant pathogens several genes play a role in regulating pathogenicity. For example, in *Magnaporthe oryzae*, Moatf1 is required for full virulence as it regulates the transcription of laccases and peroxidases to disrupt reactive oxygen species-mediated plant defense [5]. MoAP1 is another bZIP that mediates the oxidative stress response and is important for growth, development, and pathogenicity [6]. In the soilborne fungal pathogen *Fusarium oxysporum*, the bZIP HapX mediates iron homeostasis and is essential for rhizosphere competence and virulence [7].

Oomycetes, which include many plant pathogens, are fungus-like organisms that are actually evolutionarily related to brown algae and classified in the kingdom Stramenopila [8]. Currently, little is known regarding the functions and regulation of the bZIP family in oomycetes. Only Pibzp1, a bZIP family protein in *Phytophthora infestans,* has been characterized; it is known to interact with a protein kinase and is required for zoospore motility and plant infection [9]. Notably, within the DNA-binding region of Pibzp1, an asparagine residue that is conserved in other eukaryotes is substituted with cysteine, which suggests that some bZIPs in oomycetes may be functionally distinct [9]. Although the genomes of several oomycete plant pathogens have been sequenced [10–13], systematic identification and analysis of the whole bZIP family in comparison with non-oomycete organisms is currently lacking.

*Phytophthora sojae*, one of the most well-studied oomycete plant pathogens, causes stem and root rot of soybean and is responsible for serious crop losses worldwide [14]. The draft sequence of the *P. sojae* genome has greatly accelerated the study of *Phytophthora*-host interactions [15]. In this study, we developed a bioinformatics pipeline and identified a comprehensive set of bZIP family members in *P. sojae* and another eight species of oomycetes, diatoms, and fungi. In addition to the conventional bZIPs, several novel classes of bZIPs were identified, and their expansion contributed to a larger bZIP family in oomycetes, especially *Phytophthora*. In addition, phylogenies, secondary protein structures, and gene expression patterns were analyzed and compared between conventional and novel classes of bZIP candidates. These results provide important data for further functional studies of bZIPs and other transcription factor families, and increase our understanding of potential transcriptional regulatory mechanisms in oomycetes.

## Results and discussion

### Large numbers of bZIP candidates in *P. sojae*

To identify bZIP proteins encoded in the *P. sojae* genome, hidden Markov models (HMMs) of the bZIP domain were downloaded from the Pfam database [16] and used to search the 19027 predicted *P. sojae* protein sequences [10] using an e-value cutoff of 1e-5. Seventy proteins displayed matches, considerably more than the number of proteins annotated as containing “bZIP” domains in the *P. sojae* genome database (JGI v1.1, 23 genes) [10] or in the Fungal Transcription Factor Database (FTFD v1.2, 17 genes) [17]. To validate the bZIP candidates obtained using the Pfam HMMs, the 70 matching proteins were analyzed using two online tools, namely the Batch CD-search tool (CDD, in NCBI) [18] and the SMART database [19]. Based on these results, 34 proteins were confirmed to be bZIP candidates by at least one of those two tools. To identify the full set of *P. sojae* bZIP family proteins, the sequences of the bZIP domains from the 34 confirmed candidates were aligned to build a novel HMM, which was used to re-search the *P. sojae* protein sequences for further candidates. The process of rebuilding the HMM, confirming candidates with CDD and SMART, and then building a new HMM was repeated until no new proteins were identified. By this process, 45 bZIP TF candidates were obtained. A further 62 proteins were identified from the HMM predictions that were not confirmed by CDD or SMART (Additional file 1: Table S1).

Among the 45 candidates, over 30% exhibited substitutions in the conserved asparagine residue within the basic region of the bZIP domain (Additional file 1: Table S1), indicating that the *P. sojae* genome encoded some non-conventional bZIP or bZIP-like class proteins. We hypothesized that some additional bZIP proteins remained that were not detected using the above method that was originally developed for identifying conventional bZIP domains. Therefore, to search for additional variant bZIPs, the 45 full-length protein sequences were used to individually query the *P. sojae* protein sequences using the blastp program with a strict e-value (1e-40) cutoff. This search produced 34 new protein matches, 23 of which (68%) were present among the 62 sequences detected by the HMM predictions but not confirmed by CDD or SMART analyses, plus another 11 that were completely novel. bZIP domains within these 34 proteins were non-conventional and thus could not be directly predicted using CD-search or SMART. However, in 26 of the 34 proteins, bZIP domain regions could be manually identified from the HMM or blast alignments based on comparisons with conventional bZIP domains. Thus, these 26 candidates were added to the previous set of 45 proteins to form a set of 71 high quality *P. sojae* bZIP candidates for the purposes of the next steps in the analysis. The pipeline and a summary of the prediction process are listed in Additional file 1: Table S1. The gene models for the 71 high quality candidates were manually checked and errors in the predicted gene coding regions were corrected by mapping to expressed sequence tags (ESTs) [20] or according to well-characterized features of *P. sojae* introns, e.g., most introns are between 60 and 90 bp, and begin with GT and end with AG [21]. Based on the coordinates in genome scaffolds, the 71 genes were arranged and named *PsBZPc01* to *PsBZPc71* (since the transcription factor functions of the encoded proteins have not been experimentally confirmed, we termed them *P. sojae* bZIP candidates, PsBZPc). Details of these genes are provided in Additional file 1: Table S2. The lengths of encoded proteins ranged from 167 to 829 amino acids (aa), with an average of 382 aa. Introns were absent from 48 genes (68%), while 11, 7, and 5 genes had 1, 2, and >2 introns, respectively.

Due to the somewhat arbitrary nature of the cutoff used to select the 71 high quality candidates, it is possible that some false positives that lack TF function may be included among these candidates, and furthermore that some highly variant but functional bZIP TF members may have been erroneously excluded.

### Many oomycete bZIP candidates contain novel DNA-binding domains

**Figure 1:**
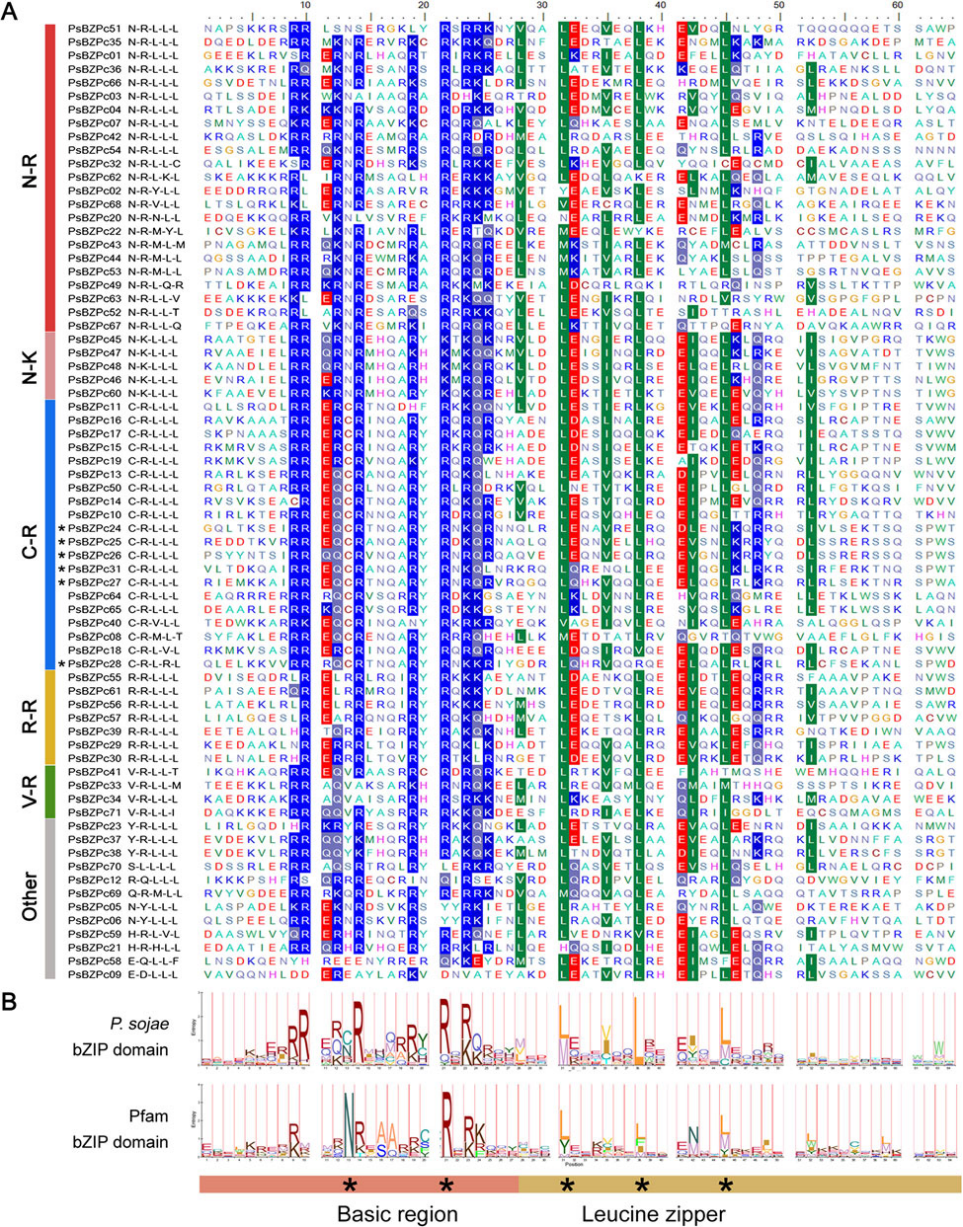
**Alignment of 71 candidate** ***Phytophthora sojae*** **bZIP domains. A**, The first two and three conserved residues in the basic regions and leucine zippers, respectively, are displayed at the right side of the protein name, e.g., “N-R-L-L-L.” Six Pibzp1 homologs are marked by asterisks. Residues with higher identities were highlighted by BioEdit [23], e.g., the basic amino acids Arg (R) and Lys (K) are highlighted in blue, the acidic amino acids Asp (D) and Glu (E) in red, and the hydrophobic amino acids Leu (L), Val (V), Ile (I), and Met (M) in green. **B**, Sequence logos of *P. sojae* and Pfam bZIP domains. The first two and three conserved sites in the basic region and leucine zipper region respectively are marked by asterisks.

Excluding PsBZPc32 (which contained a PAS domain at the C-terminal end, downstream of the bZIP domain), all *P. sojae* bZIP candidates contained one bZIP domain, and no extra functional domains were predicted in any of them (Additional file 1: Table S2). To explore the characteristics of these candidate bZIP domains, their protein sequences were compared with conventional bZIP domains available in the Pfam database (ID: PF00170) [16]. As shown in Figure 1A and B, some conserved residues between the *P. sojae* bZIP domain and the conventional Pfam bZIP domain differed, especially within the basic region, which is important for DNA sequence recognition [22]. Of the basic DNA-binding region, the most obvious difference was that the normally highly conserved Asn (N) residue was substituted with Cys (C), Arg (R), Val (V), or other amino acids in many *P. sojae* bZIP candidates.

Considering the two most highly conserved sites in the basic region of the bZIP domain, the conventional pattern of invariant residues was N-×7-R/K (termed the “N-R/K” bZIP class in this study), with N-R being the most frequent (N13 and R21 in the Pfam model; Figure 1B). In *P. sojae*, the two major bZIP classes were N-R/K/Y containing 30 proteins (43%; 23 having N-R) and C-R containing 20 proteins (28%) (Figure 1A, Additional file 1: Table S2). The bZIP previously reported in *P. infestans*[9], *Pibzp1*, is an example of the C-R class. The protein sequence of *Pibzp1* was used to search the *P. sojae* bZIPs using Blastp. Six matches were found at an e-value cutoff of 1e-20 (PsBZPc25 was the best hit, e-value of 3e-53), all of which belonged to the C-R class (Figure 1A, Additional file 1: Table S2).

bZIPs form 2:1 complexes with target DNA [3], whereby each bZIP monomer makes specific contacts with cognate DNA half-sites via their basic regions, which form continuous long α-helices with C-terminal leucine zipper regions that promote dimerization [24]. We submitted the full-length protein sequences of the 71 *P. sojae* bZIPs to the NPS@ [25] Web server to predict α-helices. Most (>60) *P. sojae* bZIP domains were predicted to form a long α-helix without any break through the core basic and leucine regions (Additional file 2: Figure S1). No significant difference was found between the N-R and non-N-R class bZIPs in the prediction results. The presence of the expected secondary structure predictions in both conventional and novel *P. sojae* bZIPs supports that these are fully functional proteins.

Previous studies have shown that residues in the bZIP basic region are associated with DNA-specific recognition during protein-nucleic acid interactions. For example in mouse, a dominant mutation within the DNA-binding domain of the bZIP TF *Maf* causes murine cataracts and results in selective alterations to DNA binding [26]. Replacement of invariant bZIP residues within the basic region of the yeast transcriptional activator GCN4 can also affect DNA-binding specificity [27]. Furthermore, an arginine to lysine substitution in the bZIP basic region of the opaque-2 transcription factor in maize abolished its specific DNA binding [28]. Therefore, the novel basic region substitutions in the presumptive DNA-binding domains of the oomycetes bZIPs may indicate that they have novel DNA binding specificities or even may interact with different kinds of molecules.

### Selective expansion of novel bZIP classes in oomycetes

To survey the distribution of these novel classes of bZIP TFs in more species, the bZIP prediction pipeline, including the building and updating of species-specific HMMs, was used to predict bZIPs in four other oomycetes (*Phytophthora ramorum*, 60 candidates; *P. infestans*, 47; *Pythium ultimum*, 37; and *Hyaloperonospora arabidopsidis*, 25), two diatoms (*Thalassiosira pseudonana*, 20; *Phaeodactylum tricornutum*, 20), and two fungi (*Fusarium graminearum*, 25; *M. oryzae*, 25). Both oomycetes and diatoms belong to the kingdom Stramenopila [14, 29], while fungi are evolutionally distant from this kingdom. Data from the prediction process are provided in Additional file 1: Table S1, and detailed sequence data and comments on all candidates can be found in Additional file 1: Table S3.

**Figure 2:**
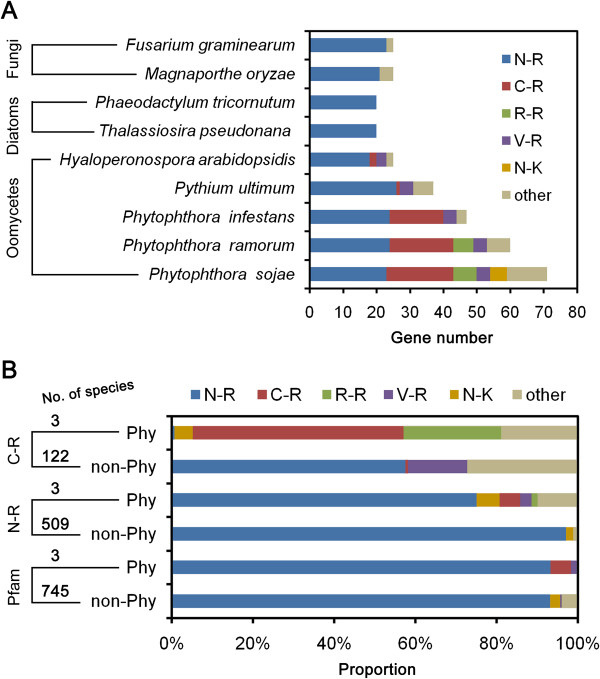
**Frequency of bZIP candidates across species. A**, Number of bZIP candidates in species of oomycetes, diatoms, and fungi. Colors represent different bZIP classes defined by the two conserved sites of basic regions. **B**, Proportions of matched genes belonging to each bZIP class. “C-R,” “N-R,” and “Pfam” on the left side indicate that the searches were performed using HMMs derived from alignments of *Phytophthora* C-R class domains, *Phytophthora* N-R class domains, and conventional Pfam bZIP domains, respectively. The numbers of species (*Phytophthora* and non-*Phytophthora*) with matching proteins are displayed.

More bZIPs were identified from oomycete species, particularly *Phytophthora* species (Figure 2A). For example, the number of bZIPs in *P. sojae* (71 candidates) was three-fold greater than that in the diatom *T. pseudonana* (20 candidates). Candidate numbers were compared in six classes of bZIPs, namely, N-R, C-R, R-R, V-R, N-K, and other (Figure 2A, Additional file 1: Tables S2 and S3). The N-R bZIP class contained around 20 members in every species. In contrast, the expansion of bZIPs in the three surveyed *Phytophthora* species was primarily due to the non-N-R classes, with the major class being C-R. Similar to the hemibiotrophic *Phytophthora* species, the necrotrophic *Py. ultimum* and biotrophic *H. arabidopsidis* also contained bZIPs other than the N-R class, but exhibited only modest expansions of those classes. As shown in Figure 2A, they contained fewer C-R class proteins. Beside the N-R class, the V-R bZIP class was found in all surveyed oomycete species, while the R-R class was found only in *P. sojae* and *P. ramorum*. In addition, N-K class bZIPs were found only in *P. sojae*. A few variant non-N-R-class bZIP candidates were identified in non-oomycete species.

### Novel classes of putative bZIP TFs are mostly restricted to oomycete species

The above predictions in nine species were based on a pipeline that began with an HMM for conventional bZIP domains. As outlined in the detailed prediction results (Additional file 1: Table S1), few additional bZIPs were identified among the non-*Phytophthora* species after the first round of HMM searches. Among oomycete species, many non-N-R class candidates were found in the first round of HMM searches, while few non-N-R class candidates were found in non-oomycete species. Thus the novel classes of putative bZIPs appeared to be mostly restricted to oomycete species.

To explore whether non-N-R classes such as C-R were more widely distributed than in the nine species examined using the pipeline, we used an alignment of C-R class bZIP domains collected from *P. sojae*, *P. ramorum*, and *P. infestans* to construct an HMM and used the HMM to query the UniProtKB protein sequence database (http://www.uniprot.org), using the “hmmsearch” tool at the HMMER Web server [30]. UniProtKB includes complete reference proteome sets from many species, though proteins from non-*Phytophthora* oomycete species hadn’t been included in UniProtKB when the searches were performed. For comparison, an alignment of N-R class domains from *P. sojae*, *P. ramorum*, and *P. infestans* was also used to create an HMM for querying UniProtKB. Based on a relaxed threshold (E-value: 1.0), the N-R HMM found 5976 matches from 512 species. The top six species were *Oryza sativa*, *Zea mays*, *Homo sapiens*, *Takifugu rubripes*, *Mus musculus*, and *Arabidopsis thaliana*, and each species had more than 120 matches (Additional file 1: Table S4). In contrast, the *Phytophthora* C-R HMM found only 430 matches from 125 species. The top six species were *P. sojae*, *P. ramorum*, *P. infestans*, *Botryotinia fuckeliana*, *Paracoccidioides brasiliensis*, and *Saccharomyces cerevisiae*, with 71, 50, 38, 11, 9, and 8 matches, respectively (Additional file 1: Table S4). Thus, the *Phytophthora* species had significantly more matches than any other species. As shown in Figure 2B, nearly all matches from *Phytophthora* species fell into non-N-R classes, e.g., C-R (52%) and R-R (24%). In the non-*Phytophthora* species, N-R (58%) and V-R (15%) classes accounted for the majority of matches, while the remainder belonged to “other.” Only one C-R class bZIP candidate (Uniprot ID: G9MT11) was found in a non-*Phytophthora* species, namely the ascomycete fungus *Trichoderma virens*. This result confirmed that the majority of novel bZIP classes were restricted to oomycete species and that some novel classes contained multiple members.

Since the sequences of oomycete bZIP domains were unusually diverse, to ensure that seeds (alignments) from *Phytophthora* did not affect the sensitivity to sequences from distant species, we used the HMM of the canonical Pfam bZIP domain (ID: PF00170) to search the UniProtKB database. This HMM identified both conventional and novel classes of bZIP domains during the above predictions (Additional file 1: Table S1). Although more matches (8059) from more species (748) were obtained with the Pfam HMM than using the *Phytophthora*-N-R HMM, no obvious differences were observed between the top matched species and domain classes. Also, the novel bZIP classes were only found in *Phytophthora* species (Additional file 1: Table S4, Figure 2B). However, one more gene was found containing a C-R class bZIP domain (Uniprot ID: E0W336, from the arthropod *Pediculus humanus corporis*). In addition, we used our pipeline to catalog all bZIP members in the genomes of *Tr. virens* and *Pe. humanus corporis*. However, only the single C-R class bZIP candidate already identified was found in each case. A phylogenetic tree was constructed based on aligned bZIP domains from bZIP candidates of *P. sojae*, *Py. ultimum*, *H. arabidopsidis*, *T. pseudonana*, and *M. oryzae*, as well as the two novel C-R domains from non-*Phytophthora* species. As shown in Additional file 3: Figure S2, the two non-oomycete C-R class candidates fell within fungal clades that mostly contained N-R bZIPs. On the other hand, all or nearly all C-R, R-R, N-K, and V-R bZIPs fell into distinct sub-clades, respectively, rather than into the N-R class (Additional file 3: Figure S2), suggesting that genes for these novel bZIP classes may have emerged early in oomycete evolution.

### The expanded bZIP family may result from widespread gene duplication

**Figure 3:**
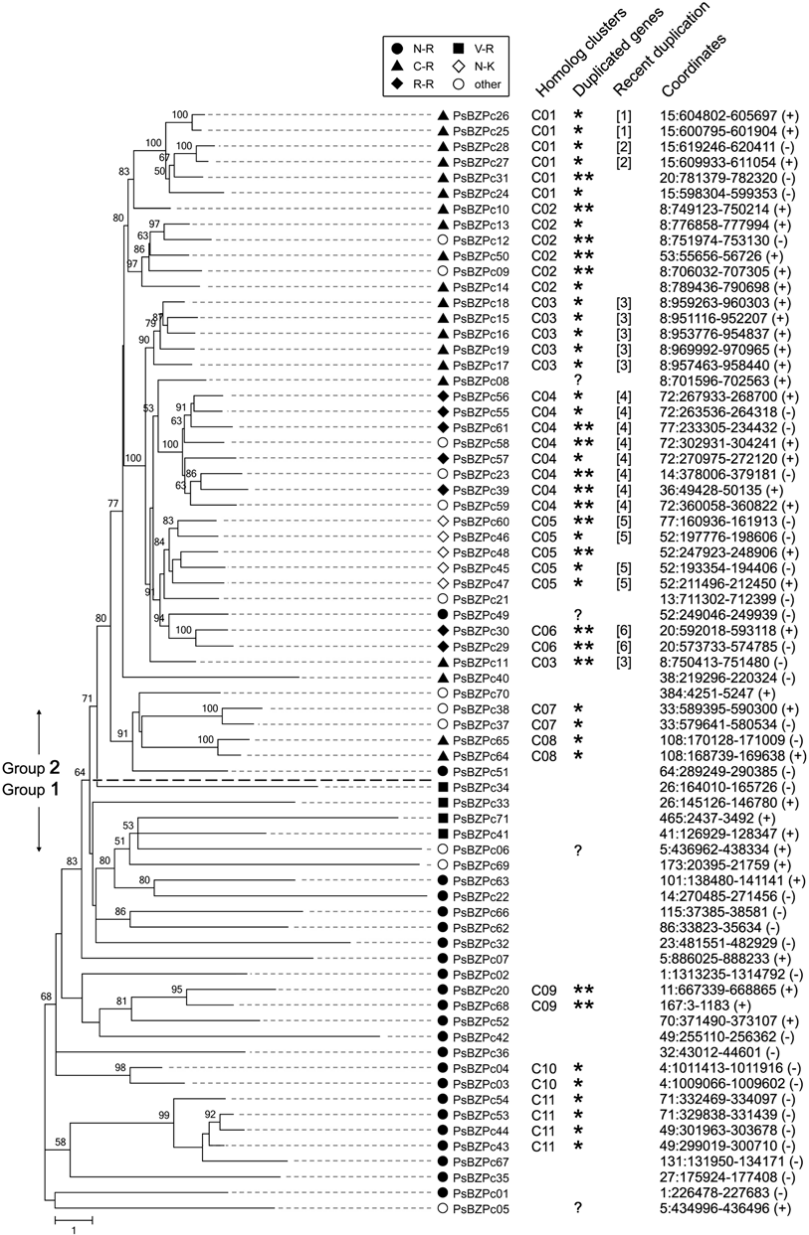
**Phylogenetic tree of** ***P. sojae*** **bZIP full length proteins.** The maximum-likelihood trees were constructed based on the full-length protein sequences; bootstrap values above 50 are shown near the tree nodes. The black dotted line divides the candidates into group 1 and group 2. Different bZIP classes are marked by the corresponding labels. Column “Homolog clusters” shows 11 homolog clusters that were grouped by all vs. all blastp searches with e-value of 1e-50 as the threshold. In the column “Duplicated genes”, “*” indicates inferred tandem duplications, “**” indicates duplications between scaffolds or two loci with >20 kb distance within scaffolds, and “?” refers to bZIPs just within 20 kb of a scaffold. In the column “Recent duplication”, five gene clusters are indicated in which the duplications were inferred to occur subsequent to the radiation of *P. sojae*, according to Additional file 5: Figure S4.

To further examine the evolution of the *P. sojae* bZIPs, we constructed phylogenetic trees either from the full length *P. sojae* bZIPs or from the bZIP domains alone, the basic regions alone, or the leucine regions alone (Figure 3, Additional file 4: Figure S3A through D). The arrangement of the different bZIP classes within the trees was generally similar irrespective of the regions of the proteins used to construct them, suggesting relatively few recombination events contributed to the origins of these proteins. As shown in Figure 3, nearly all N-R class bZIPs and all V-Rs formed a distinctive group located at the bottom of the tree (termed group 1). Within the trees constructed using full length bZIP proteins or bZIP domains (Figure 3 and Additional file 4: Figure S3A, B, respectively), many clades in group 1 had longer branch lengths, indicating more diversification of the sequences. All C-R, N-K, and R-R class bZIPs and most “other” class bZIPs formed a distinctive group located at the top of tree (termed group 2), with shorter branch lengths and a better-supported topology. Thus these bZIPs exhibited lower levels of sequence diversification and likely arose through more recent gene duplications. This inference is supported by a phylogenetic tree of full length bZIP proteins from *P. sojae*, *P. infestans* and *P. ramorum* (Additional file 5: Figure S4). This tree reveals that many bZIPs in group 2 are found in species-specific clades, indicating that duplication and diversification occurred subsequent to the divergence of the three species. In contrast, many bZIPs in group 1 are found as sets of orthologs conserved in the three species, separated by relatively long branches, indicating that the bZIPs had diverged from one another prior to speciation.

Based on all-to-all blastp searches of the 71 candidates using a 1e-50 e-value threshold, 45 (63%) of the *P. sojae* bZIPs fell into 11 groups likely resulting from recent duplications (Figure 3). After mapping bZIPs onto the genomic scaffolds, many tandem duplications and duplications within/between scaffold(s) were inferred (indicated with asterisks in Figure 3). For example, PsBZPc24 to PsBZPc28 appeared to be duplicated in tandem within scaffold_15, and additional duplicates appear to have been translocated to scaffold_20 (PsBZPc31) and scaffold_8 (PsBZPc10) (assuming the apparent translocations do not result from sequence assembly errors). Most of the genes in group 2 (37/43; 86%) are located within sub-clades that correspond primarily to genomic clusters, supporting the inference that most of the group 2 sequences result from recent gene duplications. Several of these clusters are specific to *P. sojae* (e.g., PsBZPc55-c59 and PsBZPc15-c19) (Additional file 5: Figure S4; Figure 3) indicating that the duplications occurred subsequent to the radiation of *P. sojae*. Others however include bZIPs from other *Phytophthora* species (Additional file 5: Figure S4) (e.g., PsBZPc64 and PsBZPc65; PsBZPc37 and PsBZPc38) indicating that the duplication may have occurred prior to the separation of *Phytophthora* species. In contrast to group 2, only 8 of the 28 group 1 members (29%) show evidence for gene duplications and even in those cases the presence of orthologs of those genes in other *Phytophthora* or oomycete species (Figure 3; Additional file 3: Figure S2 and Additional file 5: Figure S4) suggests that the duplications predated the radiation of those species. The majority of the group 1 members show distinctive ortholog relationships with bZIPs from other *Phytophthora* and oomycete species consistent with a more ancient origin. Since the novel bZIP classes were located in distinct phylogenetic trees and exhibited substantially more frequent recent putative gene duplications, it seems plausible that they may have been recruited for functions distinctive to oomycetes, such as infection or zoospore development.

### Zoosporangia, zoospore and cyst stages, and host infection correspond to major transcriptional shifts in bZIPs

**Figure 4:**
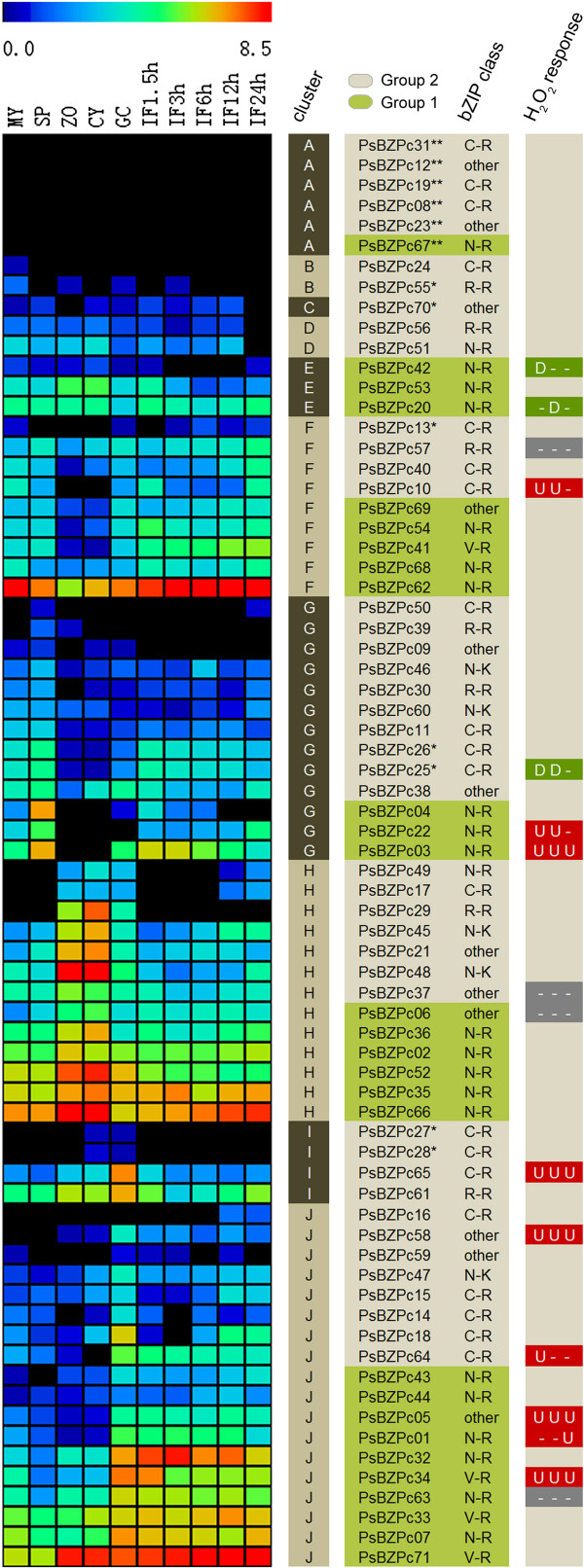
**Transcription patterns of** ***P. sojae*** **bZIP candidates.** The heat map shows gene transcription patterns during 10 stages of the life cycle: mycelia (MY), zoosporangia (SP), zoospores (ZO), cysts (CY), germinating cysts (GC), and samples from 1.5, 3, 6, 12, and 24 h post-inoculation of soybean leaves (IF1.5 h to IF24 h). The color bar represents log_2_ of gene transcripts per million reads (TPM) values, ranging from dark blue (0) to red (8.5). Black represents no expression. “**” following protein ID indicates that gene expression was not detected in all 10 libraries, and “*” denotes ambiguous data because of other identical sequences [31]. Transcript level data were collected into 11 clusters using HCL methods and MeV software, and marked in the column “cluster” (A to J). Column “H_2_O_2_ response” shows the patterns of transcriptional changes following H_2_O_2_ treatments at 30 min, 1 h, and 2 h, respectively. “U” and “D” refer to >1.2 fold elevation or -reduction respectively that are significant with a *t*-test P-value < 0.05, while “-“ means no significant change.

Transcriptome data from previous EST [20] and Digital Gene Expression (DGE) [31] analyses were used to examine expression patterns of the bZIP genes during different developmental and host-infection stages. Among 44 ESTs that mapped to bZIP genes, 34 mapped to bZIPs in group 1 (Additional file 1: Table S2), suggesting that the gene expression levels of group 1 may be higher than those of group 2. The DGE study collected extensive gene expression data using a 3′-tag protocol from 10 stages of the *P. sojae* life cycle, including five free living developmental stages and five host (soybean) infection stages [31]. Of the 71 *P. sojae* bZIP genes, the transcripts from 6 could not be detected, while the remaining 65 were detected during at least one stage (Figure 4), suggesting that few, if any, of these genes may be pseudogenes. PsBZPc23 and PsBZPc12 were the most likely to be pseudogenes as they both fell into the “other” class and also lacked any transcripts. In general, the bZIPs in group 1 were expressed more highly than those in group 2 (Figure 4), consistent with the EST data.

Varied stage-specific expression patterns were observed. Based on hierarchical clustering (HCL), 11 gene expression clusters were obtained. As shown in Figure 4, four clusters encompassing 53 genes (75%) represented the major expression patterns of *P. sojae* bZIP candidates. Cluster-F (13%) exhibited reduced transcript levels in zoospore and cyst stages. Cluster-G (18%) exhibited elevated transcript levels in zoosporangia stage and reduced transcript levels in zoospore and cyst stages. Cluster-H (18%) exhibited highly elevated transcript levels during zoospore and cyst stages. Cluster-J (25%) exhibited elevated transcript levels in germinating cysts that remained high during infection. In addition, Cluster-I with 4 members exhibited high transcript levels during cysts and germinating cysts stages. These clusters indicated that the major transcriptional shifts of bZIPs occurred during the zoosporangia/zoospore/cyst and host infection stages.

When comparing the proportions of genes in groups 1 (28 genes) and 2 (43 genes) belonging to each gene expression cluster (Figure 4), group 1 had higher proportions of genes in cluster-F (18% vs. 9%), cluster-H (21% vs. 16%), and cluster-J (36% vs. 19%); group 2 had a higher proportion in cluster-G (23% vs. 11%). However, none of these differences were statistically significant (chi-square p > 0.10). In *P. infestans*, *Pibzp1* was highly expressed during the mycelia stage, but repressed during the zoospore and cyst stages [9]. In *P. sojae*, the homologous gene *PsBZPc25* had a similar expression pattern (Figure 4).

### Several infection-associated bZIPs were also regulated following H_2_O_2_ treatment

**Figure 5:**
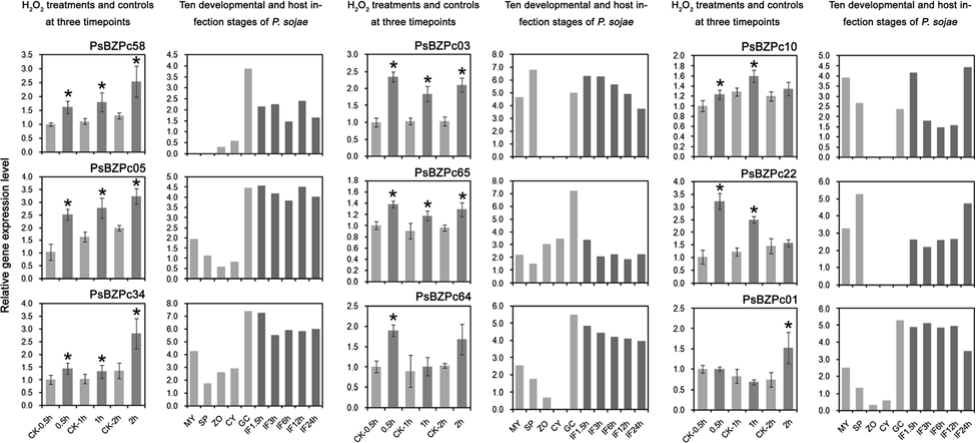
**bZIP candidates with elevated transcripts following H**_**2**_ **O**_**2**_ **treatment.** Data of controls at 30 min (CK-0.5 h) were used as reference and normalized to 1.0 using *P. sojae act*A (GeneID: 108986) as a reference. Based on the treatment-control comparisons, asterisks indicate >1.2 fold elevation or reduction respectively that are significant with a *t*-test P-value <0.05. The data were averages of three independent replicates. Error bars indicate standard deviations. Histograms showing the digital gene expression profiling data from ten developmental and infection stages are shown on the right side of the H_2_O_2_ histogram for each gene.

An oxidative burst is one of the first host plant defense responses to invasion by pathogens. Previous reports indicated that some bZIPs were associated with the response of pathogens to oxidative stress and plant signals [6, 32]. The transcription patterns of selected *P. sojae* bZIPs were measured under oxidative stress using Real-Time PCR. Liquid-V8-media-grown 2-day-old mycelia were moved to Plich media for 24 hours, and then H_2_O_2_ was added to 2 mM as a treatment while parallel cultures with no H_2_O_2_ treatment were maintained as controls. Three samples were collected from treatment and control cultures for RNA extraction at 30 min, 1 h, and 2 h post treatment. A total of 16 bZIPs, including several infection-associated and some continuously expressed ones, were selected and their transcript levels were measured by qRT-PCR (Figures 4 and 5; Additional file 6: Figure S5). As detailed in Figure 5, transcript levels of several infection-induced bZIPs, e.g., PsBZPc58, PsBZPc05, PsBZPc34, PsBZPc03, PsBZPc64, and PsBZPc01, were significantly elevated by up to 3.5 fold following H_2_O_2_ treatment at one or more time-points (P < 0.05, *t* test comparison to control). PsBZPc65 (induced in germinating cysts), PsBZPc10 (induced in germinating cysts, mycelia, and 24 h post infection), and PsBZPc22 (induced in zoosporangia and 24 h post infection) also showed significantly elevated transcript levels following H_2_O_2_ treatment. These data suggest that some infection-associated bZIPs are also regulated by oxidative stress. The other bZIPs assayed either showed small but significant reductions in transcript levels following treatment (PsBZPc42, PsBZPc25, and PsBZPc20; Figure 4; Additional file 5: Figure S4A) or were unaffected by H_2_O_2_ (PsBZPc37, PsBZPc06, PsBZPc63, and PsBZPc57; Figure 4; Additional file 5: Figure S4B).

## Conclusions

In this study, a comprehensive set of bZIP transcription factor candidates encoded in the *P. sojae* genome were compared to similarly predicted sets from four other oomycetes, two diatoms, and two fungal species. The analysis revealed an expanded number of bZIP candidates in oomycetes, especially the *Phytophthora* species, primarily due to the expansion of several novel classes of bZIP candidates. The majority of novel bZIP classes were restricted to the oomycete species. Phylogenetic and genome location analyses indicated that the increased number of genes encoding the novel bZIP classes might have resulted from widespread gene duplications during oomycete evolution.

The novel bZIP classes are characterized by substitutions in residues within the basic region that are typically conserved in other organisms, and may play an important role in DNA-binding specificity. Therefore, the novel basic region substitutions in the presumptive DNA-binding domains of the oomycetes bZIPs may indicate that they have novel DNA binding specificities or even may interact with different kinds of molecules. One novel bZIP TF from *P. infestans* that has been well characterized, Pibzp1, is a member of the novel bZIP class, C-R, and has been shown to regulate motility and infection [9].

We examined transcription of the 71 *P. sojae* candidates to explore their possible functions, including comparison between the conventional and novel bZIP classes. Transcripts were detected from the majority of candidates using DGE and qRT-PCR, suggesting that most genes, including those encoding the novel bZIPs, are not pseudogenes. Consistent with this observation, the predicted secondary structures of both conventional and novel *P. sojae* bZIPs exhibited the expected alpha helical structures spanning the basic and leucine zipper regions. Given the diverse and important roles that bZIP TFs play in eukaryotic organisms, and the conservation of the novel bZIPs among oomycete species, it would have been surprising if these genes had turned out to be pseudogenes.

The major transcriptional shifts of bZIPs occurred during the zoosporangia/zoospore/cyst and host infection stages. Interestingly, the overall gene expression level of conventional bZIP classes was higher than that of novel classes, but the significance of the differences in gene expression patterns is not clear. Several genes that were induced during pre-infection and/or early infection were also transcriptionally regulated by H_2_O_2_ treatment, suggesting that the genes regulated by these TFs might contribute to infection by protecting the pathogen from the host oxidative burst. The expression of some of the novel oomycete-specific bZIP genes in association with developmental structures specific to oomycetes (heterokont zoospores and zoosporangia [33]) suggests that these bZIPs may directly control the development of these tissues, as has been validated for Pibzp1 [9].

The *P. sojae* bZIPs presumably interact with promoter elements to exert regulatory effects on *P. sojae* physiology. Recently, several promoter elements have been identified from genome-wide analyses of genes relevant to the spore and infection stages of *P. infestans*[34, 35]. In addition, the absence of a TATA box in the oomycete core promoter and the presence of other core promoter motifs distinct from those of model organisms have been reported [36]. Future studies will be required to determine if the novel motifs identified in oomycete promoters interact with any conventional or novel bZIPs identified in this study.

In conclusion, the identification of large classes of bZIP proteins in oomycetes with novel bZIP motif variants, that are conserved and developmentally regulated and thus presumably functional, extends our knowledge of this important family of eukaryotic transcription factors. It also lays the foundation for detailed studies of the role of these proteins in development and infection in *P. sojae* and other oomycetes.

## Methods

### Prediction of bZIP candidates

Conventional HMM models of bZIP domains were downloaded from the Pfam database at pfam.sanger.ac.uk/clan/bzip [16]. Sequences of *P. sojae* (v1.1), *P. ramorum* (v1.1), *T. pseudonana* (v3.0), and *P. tricornutum* (v2.0) protein were obtained from the DOE Joint Genome Institute (JGI) database (genome.jgi.doe.gov), *P. infestans* (release of 6/15/2009), *F. graminearum* (FG3), and *M. oryzae* (MG8) from the Broad institute database (http://www.broadinstitute.org), *Py. ultimum* (DAOM BR144; pug1) from the *Pythium* Genome Database (pythium.plantbiology.msu.edu), and *H. arabidopsidis* (v8.3) from the VBI Microbial Database (eumicrobedb.org) [37].

The “hmmbuild,” “hmmscan,” and “hmmsearch” tools in the Hmmer v3.0 package and/or its online server [30] were used to format the HMM models and search the proteomes. We used 1e-5 as a cutoff e-value when searching entire genomes for bZIPs and a cutoff of 1.0 when searching UniProtKB for homologs of oomycete bZIPs. Blastp with an e-value cutoff of 1e-40 was used to search for new proteins that were identical to previous candidates following the HMM prediction. The Online Batch CD-search tool (http://www.ncbi.nlm.nih.gov/Structure/bwrpsb/bwrpsb.cgi) [18] and SMART database (smart.embl-heidelberg.de) [19] were used to validate the predicted bZIP domains; we required high quality candidates to be validated by at least one of these two tools.

### Analysis of bZIP sequences

Sequence alignments were generated using MUSCLE (http://www.drive5.com/muscle) [38]. The sequence logos of bZIP domains were created using the HMMER online tool (hmmer.janelia.org/search/hmmsearch) [30]. Phylogenetic maximum-likelihood (ML) trees were constructed using PhyML implemented in SEAVIEW (pbil.univ-lyon1.fr/software/seaview.html) [39] and graphically viewed using MEGA 5 (http://www.megasoftware.net) [40]. The full-length protein sequences of the 71 *P. sojae* bZIPs were submitted to NPS@ (using the tool “Secondary structure consensus prediction” at npsa-pbil.ibcp.fr/cgi-bin/npsa_automat.pl?page=/NPSA/npsa_seccons.html) [25] to predict α-helices.

### Analysis of gene expression patterns

*P. sojae* ESTs were collected from the Oomycete Transcriptomics Database (http://www.eumicrobedb.org/transcripts) [41], and digital gene expression profiling data were collected from the *Phytophthora* transcriptional database (PTD v1.1, phy.njau.edu.cn/ptd) and the published tag data (NCBI: GSE29651) [31]. Gene expression pattern clustering (using hierarchical clustering methods) and drawing of the heat maps were performed using MeV software (http://www.tm4.org/mev.html) [42].

### SYBR green real-time RT-PCR

Primers for qRT-PCR assay were designed by Primer3 (http://primer3.wi.mit.edu) and blastn was used to check the specificity of the sequence. The primers used in this study are listed in Additional file 1: Table S5. Total RNAs were extracted using a PureLink RNA Mini Kit (Invitrogen USA), then were transcribed to cDNA using a PrimeScript RT-PCR Kit (Takara). SYBR green real-time RT-PCR reactions used the SYBR Premix ExTaq Kit (TaKaRa Inc., Dalian, China) and the ABI PRISM 7500 fast real-time PCR system (Applied Biosystems, USA). The 7500 System Sequence Detection Software (Version 1.4) was used to analyze the relative expression levels of each sample. The *P. sojae act*A gene (GeneID: 108986) was used as a reference to standardize the expression levels. Student’s *t* test (two tails) was performed to test the significance of differences between controls and treatments.

## Availability of supporting data

All the supporting data are included as additional files.

## Electronic supplementary material

Additional file 1: Table S1: Prediction pipeline statistics of bZIPs in oomycetes, diatoms, and fungi. **Table S2** Details of predicted bZIP candidates in *P. sojae*. **Table S3** Details of predicted bZIP candidates in other species. **Table S4** Summary of HMMER search results from UniProtKB. **Table S5** Primers for qRT-PCR assay. (XLSX 92 KB)

Additional file 2: Figure S1: Predicted bZIP domains and α-helices of *P. sojae* bZIP candidates. The lines and blocks are proportional to the sequence lengths. (JPEG 527 KB)

Additional file 3: Figure S2: Phylogenetic tree of bZIP domains from different species. The maximum-likelihood tree was constructed based on the protein sequences of the bZIP domains. Bootstrap values above 50 are shown near the tree nodes. The colors and shapes of gene labels refer to their species and bZIP domain class, respectively. Two C-R class proteins found in non-oomycete species are marked by asterisks and colored by pink. (JPEG 497 KB)

Additional file 4: Figure S3: Phylogenetic trees built from different regions of *P. sojae* bZIP candidates. The trees were constructed using a maximum-likelihood method. Bootstrap values above 50 are shown at nodes. A, full-length proteins; B, bZIP domains; C, basic regions only; D, leucine zipper regions only. (JPEG 1000 KB)

Additional file 5: Figure S4: Phylogenetic tree from full length bZIP candidate proteins of *P. sojae*, *P. infestans*, and *P. ramorum*. The trees were constructed using a maximum-likelihood method. Bootstrap values above 50 are shown at nodes. The colors and shapes of gene labels refer to their species and bZIP domain class, respectively. The 1:1:1 orthologous clades and the species-specific clades are respectively marked by different colored stars. (JPEG 451 KB)

Additional file 6: Figure S5: Relative expression levels of bZIP candidate genes following H_2_O_2_ treatment. Data of controls at 30 min (CK-0.5 h) were used as reference and normalized to 1.0 using *P. sojae act*A (GeneID: 108986) as a reference. Based on the treatment-control comparisons, asterisks indicate >1.2 fold elevation or -reductions respectively that are significant with a *t*-test P-value <0.05. A, Genes with reduced transcripts at one or more time-points. B, Genes that were not significantly regulated at any time-points. Histograms showing the digital gene expression profiling data from ten developmental and infection stages are shown on the right side of the H_2_O_2_ histogram for each gene. The data were averages of three independent replicates. Error bars indicate standard deviations. (JPEG 638 KB)
