# Supplementary figures and images for: Phylogenetic and transcriptional analysis of an expanded bZIP transcription factor family in Phytophthora sojae
Source: BMC Genomics. 2013 Nov 28;14(1):839. doi: 10.1186/1471-2164-14-839 (PMC4046829; doi:10.1186/1471-2164-14-839)

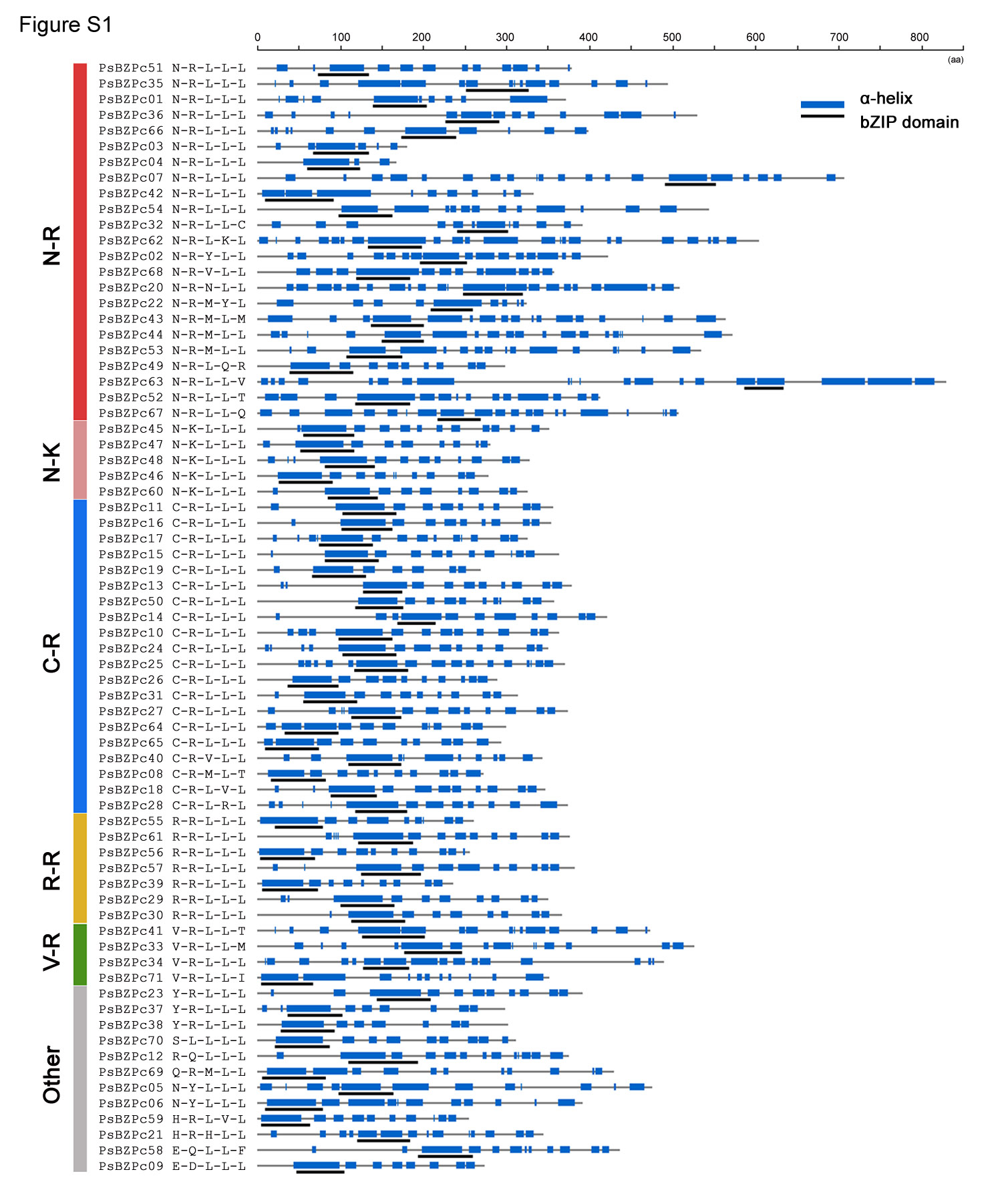

Supplement: Supplementary file 2 — Additional file 2: Figure S1: Predicted bZIP domains and α-helices of P. sojae bZIP candidates. The lines and blocks are proportional to the sequence lengths. (JPEG 527 KB) [file 12864_2013_5563_MOESM2_ESM.jpeg]

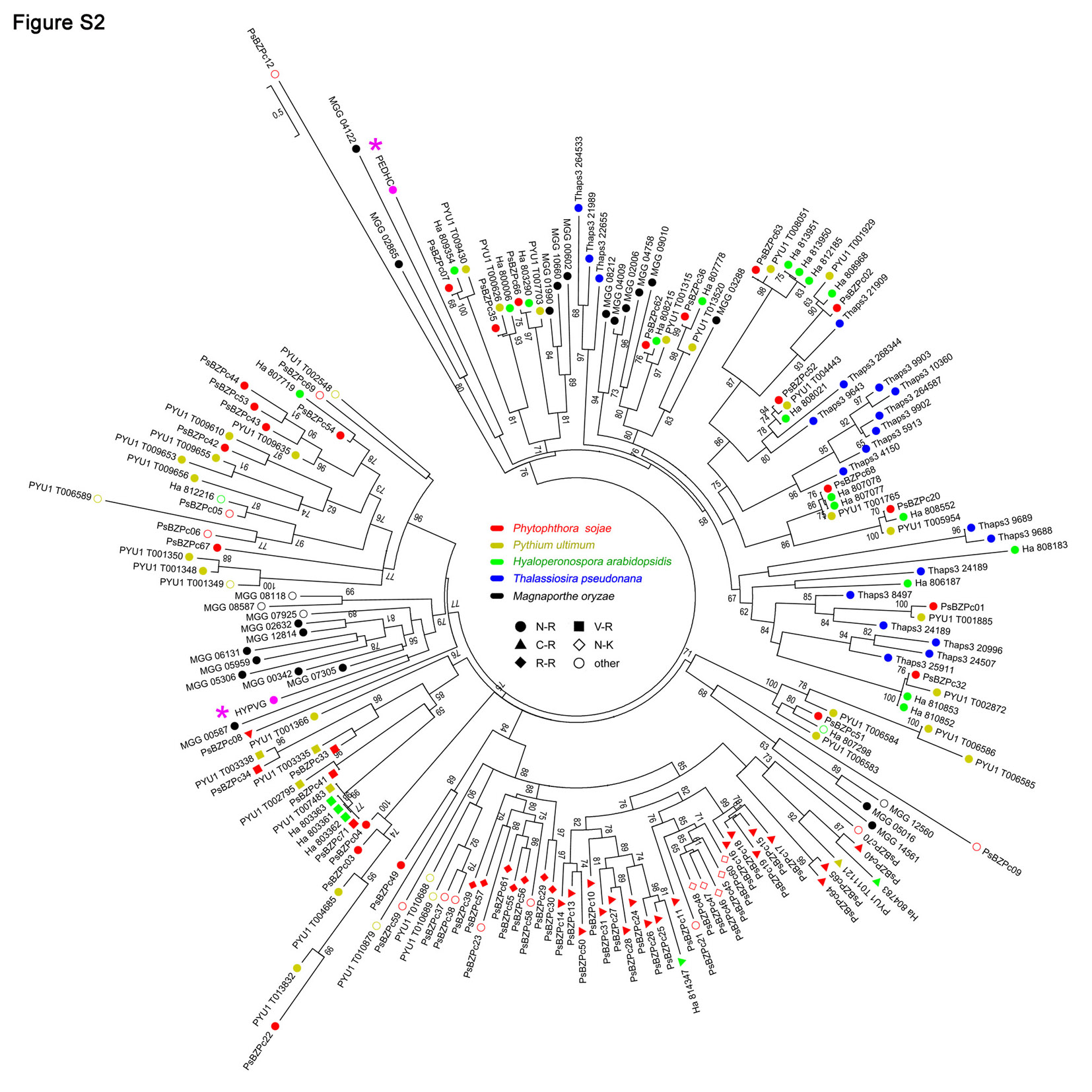

Supplement: Supplementary file 3 — Additional file 3: Figure S2: Phylogenetic tree of bZIP domains from different species. The maximum-likelihood tree was constructed based on the protein sequences of the bZIP domains. Bootstrap values above 50 are shown near the tree nodes. The colors and shapes of gene labels refer to their species and bZIP domain class, respectively. Two C-R class proteins found in non-oomycete species are marked by asterisks and colored by pink. (JPEG 497 KB) [file 12864_2013_5563_MOESM3_ESM.jpeg]

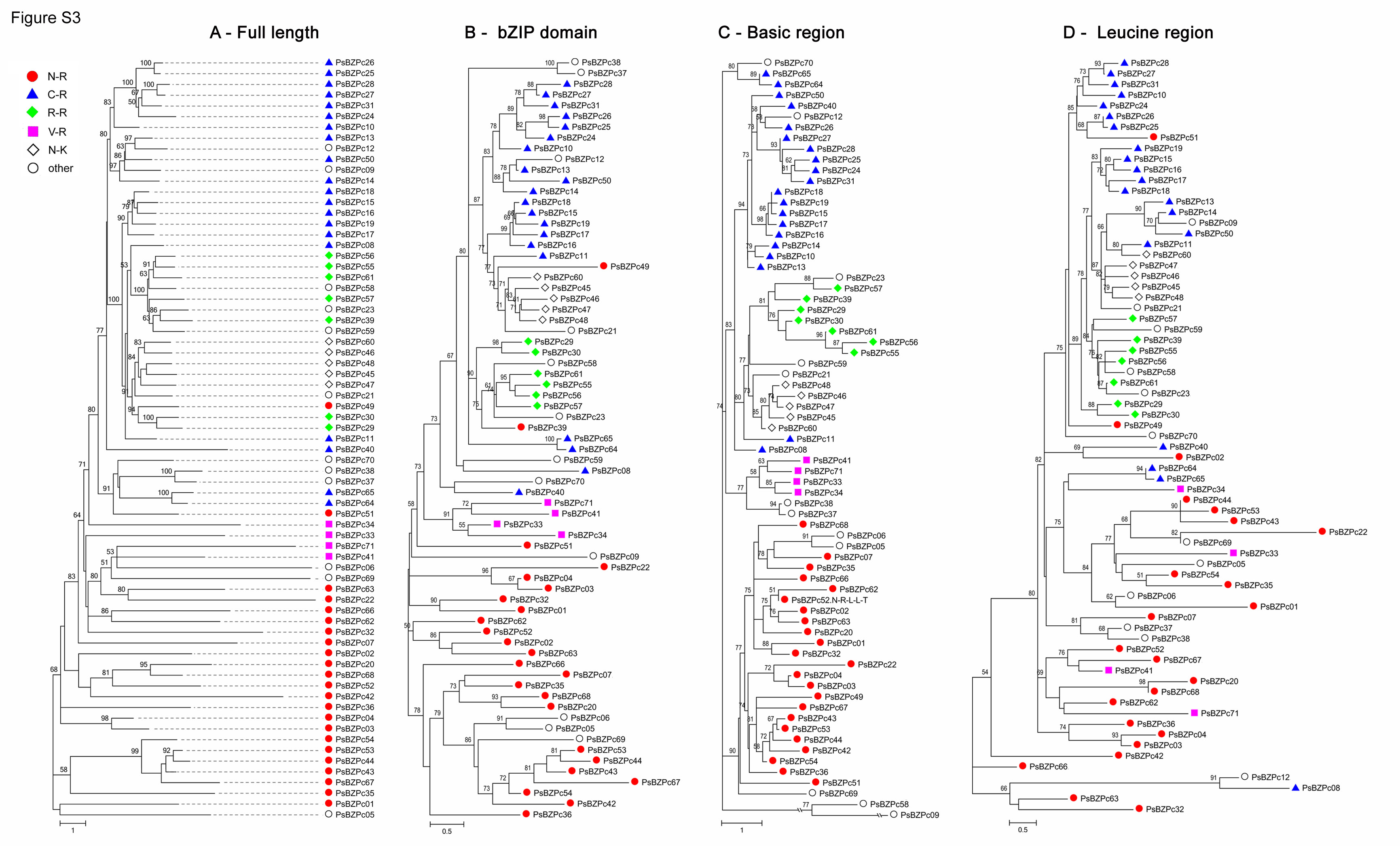

Supplement: Supplementary file 4 — Additional file 4: Figure S3: Phylogenetic trees built from different regions of P. sojae bZIP candidates. The trees were constructed using a maximum-likelihood method. Bootstrap values above 50 are shown at nodes. A, full-length proteins; B, bZIP domains; C, basic regions only; D, leucine zipper regions only. (JPEG 1000 KB) [file 12864_2013_5563_MOESM4_ESM.jpeg]

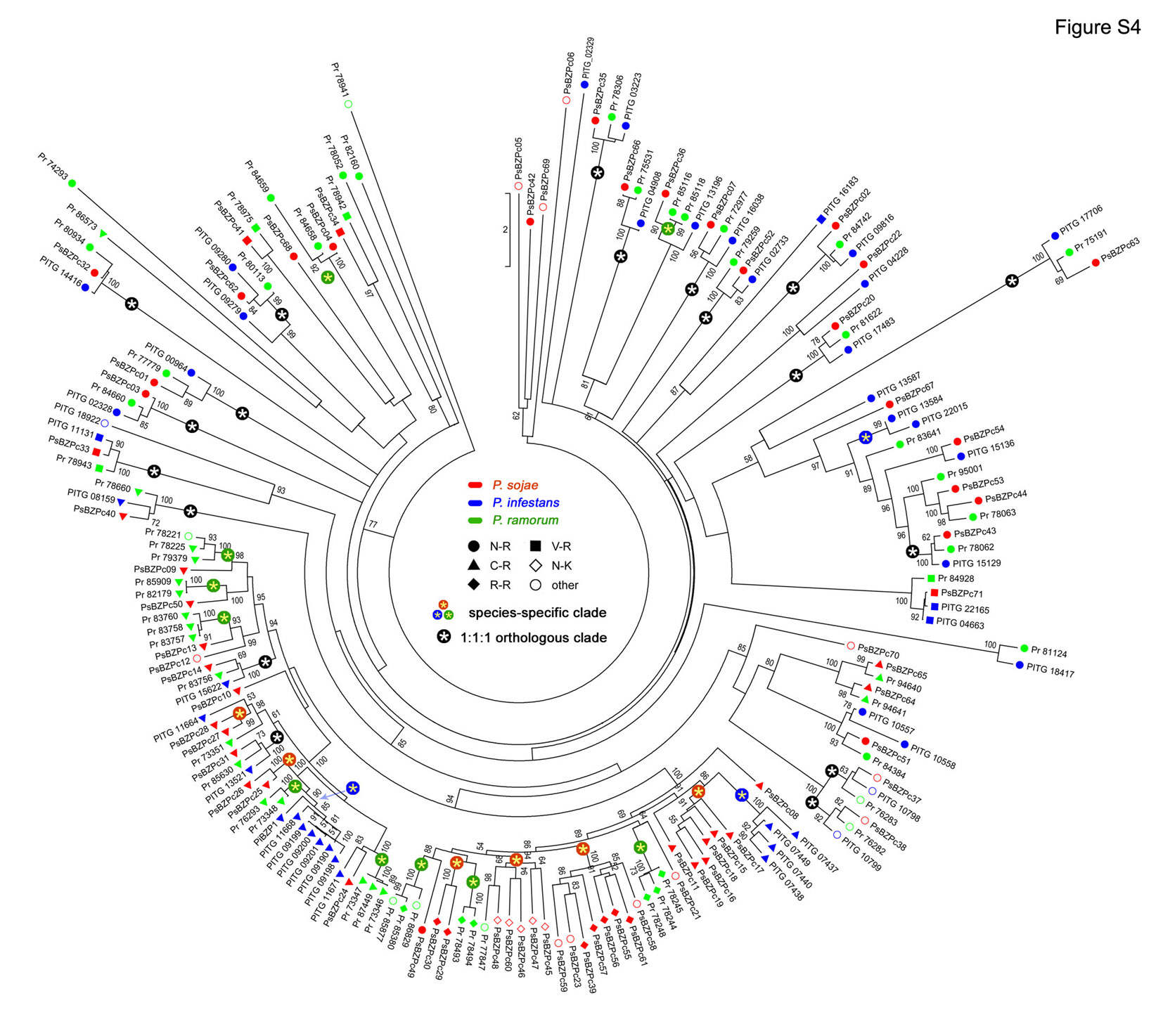

Supplement: Supplementary file 5 — Additional file 5: Figure S4: Phylogenetic tree from full length bZIP candidate proteins of P. sojae, P. infestans, and P. ramorum. The trees were constructed using a maximum-likelihood method. Bootstrap values above 50 are shown at nodes. The colors and shapes of gene labels refer to their species and bZIP domain class, respectively. The 1:1:1 orthologous clades and the species-specific clades are respectively marked by different colored stars. (JPEG 451 KB) [file 12864_2013_5563_MOESM5_ESM.jpeg]

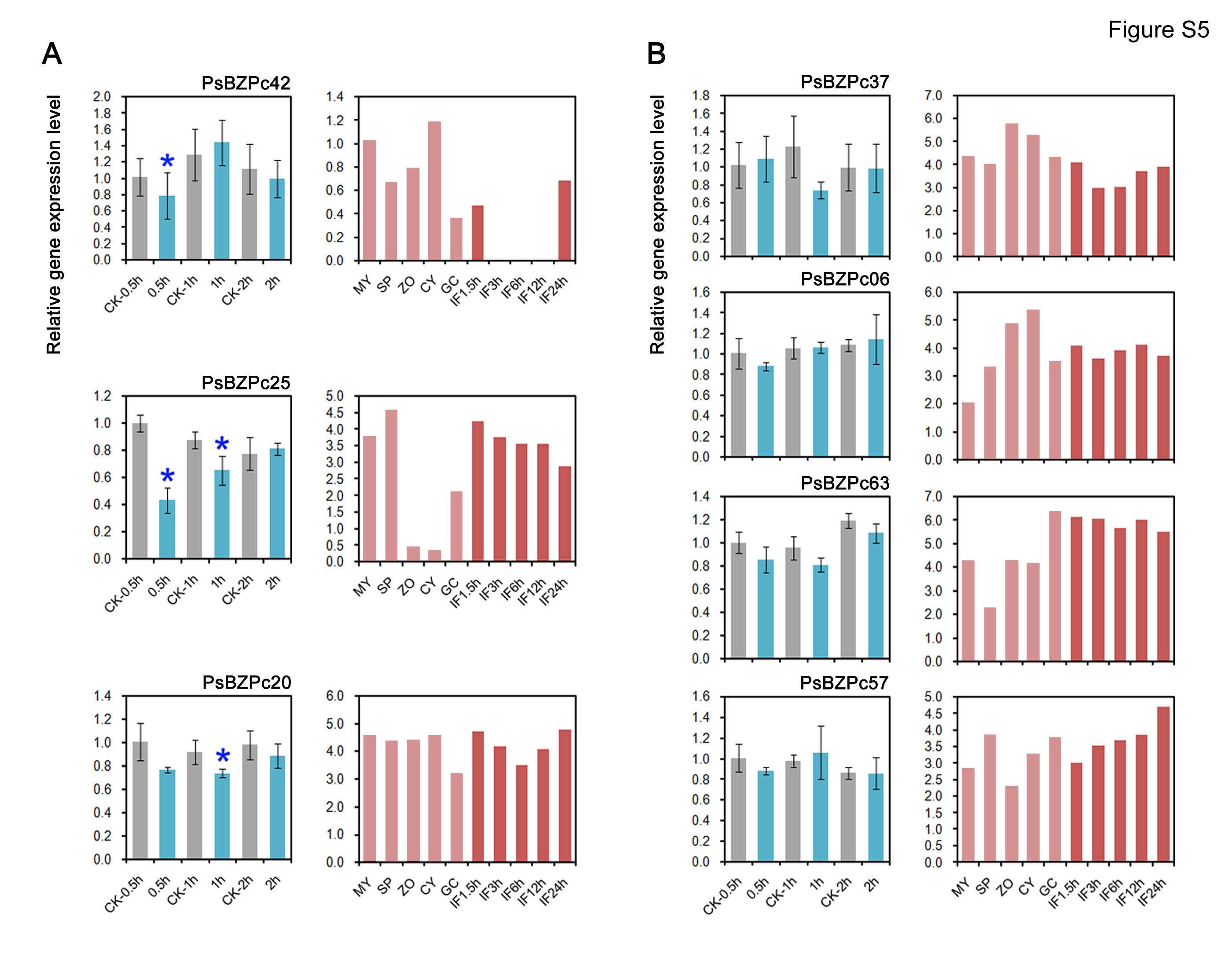

Supplement: Supplementary file 6 — Additional file 6: Figure S5: Relative expression levels of bZIP candidate genes following H2O2 treatment. Data of controls at 30 min (CK-0.5 h) were used as reference and normalized to 1.0 using P. sojae actA (GeneID: 108986) as a reference. Based on the treatment-control comparisons, asterisks indicate >1.2 fold elevation or -reductions respectively that are significant with a t-test P-value <0.05. A, Genes with reduced transcripts at one or more time-points. B, Genes that were not significantly regulated at any time-points. Histograms showing the digital gene expression profiling data from ten developmental and infection stages are shown on the right side of the H2O2 histogram for each gene. The data were averages of three independent replicates. Error bars indicate standard deviations. (JPEG 638 KB) [file 12864_2013_5563_MOESM6_ESM.jpeg]
